# Effects of Oral Cavity Stem Cell Sources and Serum-Free Cell Culture on Hydrogel Encapsulation of Mesenchymal Stem Cells for Bone Regeneration: An In Vitro Investigation

**DOI:** 10.3390/bioengineering11010059

**Published:** 2024-01-08

**Authors:** Premjit Arpornmaeklong, Supakorn Boonyuen, Komsan Apinyauppatham, Prisana Pripatnanont

**Affiliations:** 1 Faculty of Dentistry, Thammasat University-Rangsit Campus, Pathum Thani 12121, Thailand; komsanap@tu.ac.th; 2 Department of Chemistry, Faculty of Science and Technology, Thammasat University-Rangsit Campus, Pathum Thani 12121, Thailand; supakorn@tu.ac.th; 3 Faculty of Dentistry, Prince of Songkla University, Songkhla 90110, Thailand; prisana.p@psu.ac.th

**Keywords:** adipose stem cells, buccal fat pad, dental pulp stem cells, hydrogel cell encapsulation, periodontal ligament stem cells, serum-free condition, oral cavity stem cells, oral and maxillofacial osteogenesis and bone regeneration

## Abstract

Introduction: To develop a stem cell delivery model and improve the safety of stem cell transplantation for bone regeneration, this study aimed to determine the effects of stem cell sources, serum-free cell culture, and hydrogel cell encapsulation on the growth and osteogenic differentiation of mesenchymal stem cells (MSCs) from the oral cavity. Methods: The study groups were categorized according to stem cell sources into buccal fat pad adipose (hBFP-ADSCs) (Groups 1, 4, and 7), periodontal ligament (hPDLSCs) (Groups 2, 5, and 8), and dental pulp-derived stem cells (hDPSCs) (Groups 3, 6, and 9). MSCs from each source were isolated and expanded in three types of sera: fetal bovine serum (FBS) (Groups 1–3), human serum (HS) (Groups 4–6), and synthetic serum (SS) (StemPro™ MSC SFM) (Groups 7–9) for monolayer (m) and hydrogel cell encapsulation cultures (e). Following this, the morphology, expression of MSC cell surface antigens, growth, and osteogenic differentiation potential of the MSCs, and the expression of adhesion molecules were analyzed and compared. Results: SS decreased variations in the morphology and expression levels of cell surface antigens of MSCs from three cell sources (Groups 7m–9m). The levels of osteoblastic differentiation of the hPDLSCs and hBFP-ADSCs were increased in SS (Groups 8m and 7m) and the cell encapsulation model (Groups 1e, 4e, 7e–9e), but the promoting effects of SS were decreased in a cell encapsulation model (Groups 7e–9e). The expression levels of the alpha v beta 3 (ITG-αVβ3) and beta 1 (ITG-β1) integrins in the encapsulated cells in FBS (Group 1e) were higher than those in the SS (Group 7e). Conclusions: Human PDLSCs and BFP-ADSCs were the optimum stem cell source for stem cell encapsulation by using nanohydroxyapatite–calcium carbonate microcapsule–chitosan/collagen hydrogel in serum-free conditions.

## 1. Introduction

Mesenchymal stem cell (MSC) transplants in skeletal defects play essential roles of enhancing bone regeneration and vascularization. The transplanted cells can differentiate into functional cells and secrete an extracellular matrix or cytokines and growth factors to stimulate osteogenic and angiogenic cells [1,2,3]. As a result, they promote the tissue regeneration capacity of host bone and bone substitutes [4,5,6,7]. A direct role of the transplanted MSCs in bone regeneration is demonstrated by the identification of cells and proteins of a human origin in the matrix of the newly formed bone in animal models [4,8]. Additionally, the importance of the paracrine effects is further highlighted by the biologic effects of the exosomes from MSCs that could promote osteogenic differentiation and bone regeneration, and attenuate the inflammatory response [1,2,3,9]. The role of a stem cell transplant in enhancing the regenerative capacity of host bone become more essential in elderly and medically compromised patients with osteoporosis, large bone defects, and nonunion fractures, etc. [1,6,10,11,12].

However, the isolation and expansion of MSCs in a culture medium with fetal bovine serum (FBS) exposed the expanded cells to animal-derived proteins that could transfer pathogens such as mycoplasma, virus, and prion to the recipients [13,14], and stimulate inflammation [15]. Additionally, the variability of various growth factors, hormones, and protein contents in FBS could affect cell behaviors and the consistency of the experiment outcomes [13,16,17]. In contrast, synthetic sera (SS) provide consistencies in compositions and efficiency that can improve the quality, predictability, lot-to-lot consistency, and biological safety of stem cell transplants [18,19]. Accordingly, the use of an SS cell culture model is an empirical step to increase the predictability and safety of stem cell expansion and transplantation following good manufacturing practice (GMP) regulations [16,20].

Mesenchymal stem cells are multipotent stem cells found in various tissues. The oral cavity contains multiple sources of MSCs, namely, the teeth, gingival tissue, and buccal fat pads, which can differentiate into osteoblasts and promote bone regeneration [7,21,22]. The advantages of MSC transplantation are that MSCs are well characterized and can differentiate into cells in a mesodermal lineage and secrete several bioactive molecules [3,9,23,24]. Human periodontal ligament stem cells (hPDLSCs) can regenerate cementum and periodontal ligament, and are a potential stem cell source for periodontal tissue regeneration [7,25]. Human dental pulp stem cells (hDPSCs) can differentiate into osteoblasts and regenerate newly formed bone in bony defects [24,26]. A buccal fat pad (BFP) is a great source of adipose-derived MSCs (BFP-ADSCs) from the oral cavity [11,27]. In FBS, the osteogenic differentiation potential of ADSCs tends to be stronger than that of hPDLSCs and hDPSCs in a monolayer cell culture [28,29].

Human BFP (hBFP) is a fat mass collected in a thin capsule extending underneath the cheek bone. The availability of adipose tissue, easy access to stem cell sources in all age groups, and limited donor site morbidities via minimally invasive surgical procedures allow the hBFP to be a stable and accessible source of MSCs [30,31]. The lower part of the hBFP is easily reached and safely removed during surgical removal of an impacted maxillary third molar or through a minimal surgical incision at the vestibule distal to the second maxillary molar [10,11,27]. hBFP-ADSCs can differentiate into osteoblasts and promote bone regeneration [10,11,31,32]. In comparison to adipose tissues from abdominal and hip fats, the MSC phenotypes of hBFP-ADSCs from different sources are comparable, but the proliferation rate and osteogenic potential of hBFP-ADSCs are higher than those of ADSCs from other sources. In addition, hBFP-ADSCs express the cell surface markers of endothelial cells, namely, CD34 and CD146 [33].

Hydrogel cell encapsulation provides a microenvironment supporting stem cell viability and functions of the transplanted cells, and carries bioactive molecules to the defect sites [34,35]. Chitosan/collagen composite hydrogel provides an osteoconductive matrix and porous microstructure supporting the growth and osteogenic differentiation of mesenchymal stem cells [36]. Moreover, by incorporating nanocalcium phosphate particles, β-tricalcium phosphate (bTCP), and hydroxyapatite (HA), and calcium carbonate (CaCO_3_) microcapsules in the chitosan/collagen matrix it is possible to improve the microstructure and mechanical and physical properties of the hydrogels that promote the growth and osteogenic differentiation of the encapsulated cells [35,37,38]. It has been reported that a thermosensitive nanoHA (nHA)–CaCO_3_–chitosan/collagen hydrogel is a biocompatible and osteoconductive hydrogel and a delivery vehicle for bioactive molecules, such as the plant flavonoid quercetin [37]. In a FBS cell culture, the porous architecture, permeability, and swelling capacity of the hydrogel promotes the growth and osteogenic differentiation of encapsulated MSCs [35,37].

Nevertheless, to introduce a thermosensitive nHA–CaCO_3_–chitosan/collagen hydrogel [37] as a vehicle for stem cell delivery in a serum-free condition to improve the safety and consistency of the stem cell transplant for bone regeneration, the following factors must be considered, since they can influence stem cell behavior: the stem cell source, the cell transplantation method, the properties of the hydrogels, and different serum formulas [13,20,23]. The current study aimed to determine optimal stem cell sources from the oral cavity for stem cell expansion and hydrogel cell encapsulation in a synthetic serum (SS). Thus, the MSC characteristics, growth, and osteogenic differentiation of hBFP-ADSCs, hPDLSCs, and hDPSCs in the selected SS and FBS in monolayer and hydrogel cell encapsulation culture models were investigated and compared. Furthermore, the levels of cell adhesion molecules on the hydrogel in the SS and FBS cell cultures were examined. As a result, the potential effects of stem cell sources from the oral cavity, the selected SS, hydrogel encapsulation, and adhesion molecules on stem cell delivery to promote bone regeneration in serum-free conditions were elucidated. It was hypothesized that the proposed cell–hydrogel encapsulation model with optimal stem cell sources from the oral cavity in the SS would improve the quality and safety of the transplanted cells and promote bone regeneration.

## 2. Materials and Methods

### 2.1. Compliance with Ethical Standards

This study was approved by the human subject ethics board of Thammasat University, the Ethical Review Sub-Committee Board for Human Research Involving Sciences, No. 3 (Protocol number 164/2563), and was conducted in compliance with the Helsinki Declaration of 1975, as revised in 2013. Then, all patients provided their informed consent to participate in the study. Patients signed an informed consent form before undergoing surgical procedures.

### 2.2. Sample Collection

**Figure 1:**
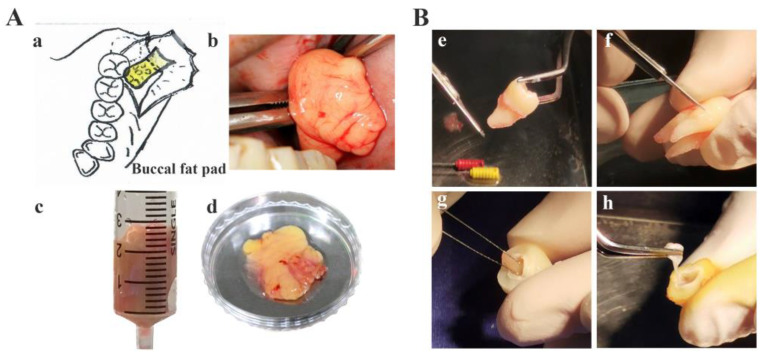
Sample collection from human buccal fat pad (**A**(**a**–**d**)) (**a**) an illustration of a surgical exposure for the removal of the impacted third molar (an outline) and the extrusion of hBFP (represented by a yellow pad) from the vestibule distal to the second molar and (**b**–**d**) the harvested hBFPs for hBFP derived adipose stem cells (hBFP-ADSCs), dental tissue (**B**(**e–h**)) (**e**,**f**)) periodontal ligament (hPDL) and (**g**,**h**) dental pulp (hDP) for hPDL and hDP stem cells (hPDLSCs and hDPSCs) respectively.

The human buccal fat pad (hBFP) and wisdom teeth were obtained from patients aged 18–25 years old who underwent surgical removal of the impacted teeth at the Dental Clinic, Thammasat University Hospital. The patients were healthy, did not take any medications, and did not have any signs of inflammation or infection at the impacted teeth. The BFP was harvested from 6 donors. Following surgical removal of the impacted upper third molars, the distal portion of the hBFP was harvested through a vestibular incision distal to the third molars following an upward blunt dissection [39]. The dental tissues were obtained from a total of 20 wisdom teeth from 10 patients [40,41] (Figure 1).

### 2.3. Human Buccal Fat Pad, Periodontal Ligament, and Dental Pulp Isolation and Expansion

In brief, the hBFP [31], periodontal ligament [40], and dental pulp tissues [41] were washed in normal saline, minced, and digested in enzyme solutions, namely, hBFP in 0.1% type I collagenase [42] and periodontal tissues and dental pulp in 3 mg/mL collagenase type I and 4 mg/mL dispase (Gibco, Thermo Fischer Scientific, Waltham, MA, USA) [41]. Then, the dissociated cells were cultured in growth medium in a humidified incubator at 37 °C under 5% CO_2_. The medium was changed on day 5 after seeding and every 2–3 days thereafter. At 80% confluence, cells were trypsinized and seeded at 5 × 10^3^ cells/cm^2^ in growth or osteogenic media supplemented with the designated sera according to study groups for expansion and investigation. Growth medium comprised of DMEM-F12 (Corning, Corning, NY, USA), 15% serum, and 1% Antibiotic-Antimycotic (all from Gibco). Osteogenic medium was a growth medium supplemented with 50 µg/mL ascorbic acid, 5 mM β-glycerophosphate, and 100 nM dexamethasone (All from Sigma–Aldrich, St Louis, MO, USA) [10,38,41,43,44].

### 2.4. Types of Sera and Cell Batches

The expanded cells were cultured in growth media supplemented with different sera according to the study groups: 15% fetal bovine serum (FBS) (Gibco), 15% human serum (HS) (Sigma–Aldrich), and 15% synthetic serum (SS) (StemPro™ MSC SFM, Gibco). For the SS group, cell culture plates were precoated with CELLstart™ Substrate (Gibco) at a concentration of 1:200 and 10 mL per 75 cm^2^ for 1 h before cell seeding following the manufacturer’s instructions. Culture media were changed every 2–3 days.

To generate cell batches, the expanded cells from each donor at passage 1 at 80% confluence were collected and frozen. Afterward, the frozen cells from three donors were thawed and pooled as one batch and expanded in the designated sera into passages 3–5 for the investigations. Cells were seeded at 5 × 10^3^ cells/cm^2^ for expansion and investigations. Growth medium comprised DMEM/F-12 (Corning, Corning, NY, USA), 1% penicillin, 0.1% fungizone (Gibco), and 15% serum (FBS, HS, or SS). The osteogenic medium was growth media supplemented with 50 mM ascorbic acid, 10 mM β-glycerophosphate, and 100 nM dexamethasone (Sigma-Aldrich) [40].

### 2.5. Study Groups

There were 9 study groups. Groups of samples were categorized into three sets: Set I—cell sources, hBFP-ADSCs, hPDLSCs, and hDPSCs; Set II—types of sera, FBS, HS, and SS; and Set III—cell culture models, monolayer cell culture (Mono, m), and hydrogel cell encapsulation (Encap, e) (Table 1).

### 2.6. Hydrogel Cell Encapsulation

An in-house thermosensitive 2% (*w*/*v*) calcium carbonate microcapsule—2% (*w*/*v*) nano hydroxyapatite—4:1 (*w*/*w*) chitosan/collagen (CaCO_3_-nHA-Ch/Col) hydrogel was used. In brief, 2% (*w*/*v*) hydroxyapatite nanoparticles and 2% (*w*/*v*) calcium carbonate microcapsules (Sigma-Aldrich) were added to a 4:1 (*w*/*w*) chitosan/collagen matrix (Sigma-Aldrich/Koken, Tokyo, Japan) with 10% (*w*/*v*) beta-glycerophosphate (Sigma-Aldrich) on ice under stirring. The ingredients of the hydrogel were pre-sterilized under a UV lamp for 30 min, and the preparation was performed in a cell culture hood using aseptic technique [35,38]. The expanded cells at passages 3–5 were added to the CaCO_3_–nHA–Ch/Col hydrogel at a concentration of 1 × 10^6^ cells/mL at 4 °C. Then, 300 µL of the hydrogel–cell suspension/well was added to a 24-well cell culture plate and incubated in a humidified incubator at 37 °C and 5% CO_2_ for 10 min; subsequently, 0.5 mL of growth medium was added to each well. Afterward, the culture medium was changed to a growth or osteogenic medium with the designated sera according to the study groups. The culture medium was changed every 2–3 days [38].

### 2.7. Flow Cytometry Analysis of Cell Surface Antigens

Flow cytometry analysis was performed to examine the expression of characteristic cell surface antigens of MSCs, namely, CD73, CD90, and CD105, and hematopoietic stem cells, namely, CD45, CD34, CD11b, CD19, and HLA-DR, using the BD Stemflow™ Human Mesenchymal Stem Cell Analysis Kit (BD Biosciences, San Jose, CA, USA) following the manufacturer’s instructions. The staining was detected and analyzed at 20,000 events per sample using fluorescent activated cell sorting (FACSARIAIII, Becton Dickinson, Franklin Lakes, NJ, USA) and BD FACSDiva Software Version 8.0 (Becton Dickinson) [40,44,45].

### 2.8. Cell Proliferation

hBFP-ADSCs, hPDLSCs, and hDPSCs were seeded in 6-well cell culture plates at 2 × 10^3^ cells/cm^2^ per well in 2 mL of growth medium. On Days 1, 3, 5, 7, and 9 after cell seeding, the cells were trypsinized and counted using hemocytometers (BLAUBRAND^®^ Neubauer pattern counting chamber, Sigma-Aldrich), and then growth curves were constructed. The number of samples was 4 per group at each investigation time (*n* = 4) [46].

### 2.9. Cell Viability and Live/Dead Cell Staining Assays

For the cell viability assay, cells were seeded on a 96-well cell culture plate at 3 × 10^3^ cells per well in growth media according to the study groups. Then, after 72 h of incubation, cell viability was determined using a CellTiter 96^®^ AQueous One Solution Cell Proliferation Assay (Promega, Madison, WI, USA) according to the manufacturer’s instructions. The optical density (OD) was read at 490 nm using a microplate reader (Varioskan Flash, Thermo Fisher Scientific).

For a live/dead cell staining assay, the encapsulated cells were stained with a LIVE/DEAD™ Viability/Cytotoxicity Kit for mammalian cells (Thermo Fisher Scientific) following the manufacturer’s instructions and examined under a confocal laser scanning microscope (CLSM) (Nikon ECLIPSE, Nikon, Melville, NY, USA) [38,47].

### 2.10. Alkaline Phosphatase and Alizarin Red Staining

Cells in osteogenic medium on Day 14 were fixed in 4% paraformaldehyde (Sigma-Aldrich) for 5 min. For alkaline phosphatase (ALP) staining, cells were stained with a BCIP/NBT liquid substrate system (Sigma-Aldrich) at RT in the dark for 10 min, and for alizarin red S staining, cells were incubated in 40 mM alizarin red solution (pH 4.2) for 20 min (Sigma-Aldrich). Then, the stained cells were washed in DI water and observed under an inverted light microscope (Evos XL core, Gibco) [40].

### 2.11. Measuring Total Protein Levels and Alkaline Phosphatase Activity

Cells in osteogenic medium on Day 14 were lysed in 1% Triton X-100 in PBS and centrifuged at 400× *g* for 5 min (Heraeus Labofuge 400R, Thermo Fisher Scientific). Then, the supernatants (cell lysate solutions) and cell pellets were collected and kept at −80 °C for further analyses. Subsequently, the protein content in the cell lysate solutions was measured using a Bio-Rad DC Protein Assay Kit (Bio-Rad Laboratories, Hercules, CA, USA) following the manufacturer’s instructions. Then, the levels of ALP activity in total protein cell lysate solutions were measured using Alkaline Phosphatase Yellow Liquid Substrate for ELISA (Sigma-Aldrich) following the manufacturer’s instructions [40].

### 2.12. Measuring Calcium Levels in the Extracellular Matrix

The cell pellets obtained from the cell lysis procedure were washed in PBS and incubated in 0.5 M hydrochloric acid at 37 °C for 12 h. Then, the supernatants were collected to measure the calcium content using a Calcium Colorimetric Assay Kit (Biovision, Milpitas, CA, USA) following the manufacturer’s instructions [40].

### 2.13. Measuring Osteocalcin Levels in Culture Medium

For levels of osteocalcin in the culture medium, on Day 14, the osteogenic medium was changed to growth medium with 1% FBS or 1% SS according to the groups of the study, and 300 µL/well was added to a 24-well cell culture plate and then incubated for 24 h. Then, the culture medium was collected for measuring levels of osteocalcin using the Takara Bio Osteocalcin ELISA Kit (TAKARA Bio, San Jose, CA, USA) following the manufacturer’s instructions. The optical density was read in duplicate using a microplate reader (Varioskan Flash).

Afterward, the levels of ALP activity, calcium content, and osteocalcin were normalized by the amount of total protein content of the same samples and reported as nanomolar p-nitrophenol, ng calcium content, and ng osteocalcin per milligram total protein content [38,40,48].

### 2.14. Quantitative Real-Time Reverse-Transcription Polymerase Chain Reaction (qRT-PCR)

Quantitative real-time reverse-transcription polymerase chain reaction (qRT-PCR) was performed to determine the expression levels of the osteoblast-associated gene Runx2, bone morphogenetic protein 2 (BMP2), and bone gamma-carboxyglutamate protein (BGLAP) as markers of the early, middle, and late stages of osteoblastic differentiation [49], and the expression levels of the alpha v beta 3 (ITG-αVβ3) and beta 1 (ITG-β1) integrins were examined to demonstrate the expression levels of the adhesion molecules (QuantiStudio 3, Applied Biosystems, San Francisco, CA, USA). Cells were disrupted using TRIzol (Invitrogen, Thermo Fisher Scientific). mRNA was extracted using a PureLink RNA Mini Kit (Invitrogen), and cDNA was synthesized using Superscript III Reverse Transcriptase (Invitrogen), 1000 ng mRNA/20 µL cDNA. Then, qRT-PCR was performed using TaqMan Gene Expression Master Mix and 20Target primer and Probe (Applied Biosystems, San Francisco, CA, USA). The primers were as follows: Runx2 (Hs01047973_m1; FAM), BMP2 (Hs00154192_m1; FAM), BGLAP (Hs01587814_g1; FAM), ITG-αVβ3 (Hs00543530_m1; FAM), and ITG-β1 (Hs01127536_m1; FAM). Then, the levels of gene expression were analyzed using the delta–delta CT method and normalized to the expression levels of GAPDH (Hs02786624_g1; FAM). Afterward, the expression levels of osteoblast-associated genes and integrins were reported as fold changes relative to the expression levels of hBFP-ADSCs on cell culture plates in osteogenic medium with FBS (FBS-OS, hBFP-ADSCs-OS) as a control group and the hydrogel-encapsulated hBFP-ADSCs in synthetic serum (SS-OS), respectively [49,50,51,52].

### 2.15. Statistical Analysis

The data are presented as the mean ± standard deviation (SD) (mean ± SD) based on an assessment of 3–5 samples. When the data were normally distributed, the differences among groups were analyzed using one-way analysis of variance (ANOVA), and multiple comparisons were made with the Sheffe or Dunnett T3 test. For non-normally distributed data, the Kruskal–Wallis and Dunn–Bonferroni post hoc tests were used for multiple comparisons. A significant difference was determined at a *p* value of <0.05. The analyses were performed using Prism Statistical Software for Mac Version 10 (GraphPad Software, San Diego, CA, USA).

## 3. Results

### 3.1. Cell Morphology and Expression of MSC Surface Antigens

**Figure 2:**
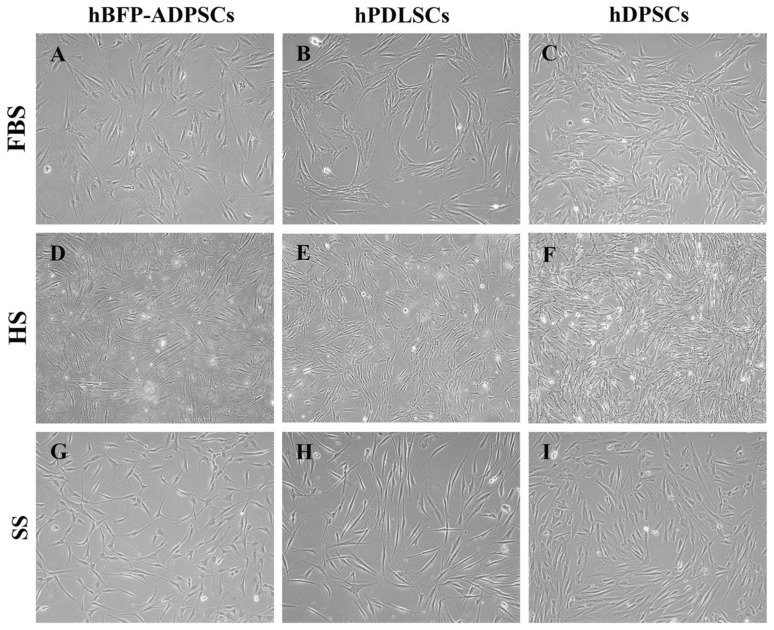
Morphology of mesenchymal stem cells from the oral cavity, human buccal fat pad-derived adipose stem cells (hBFP-ADSCs) (**A**,**D**,**G**), periodontal ligament stem cells (hPDLSCs) (**B**,**E**,**H**), and dental pulp stem cells (hDPSCs) (**C**,**F**,**I**) in fetal bovine serum (FBS) (**A**–**C**), human serum (HS) (**D**–**F**), and synthetic serum (SS) (**G**–**I**) (magnification ×10).

The spindle-shaped cell morphology and expression of MSC surface antigens varied with the stem cell sources and types of sera. In growth media with FBS and HS (Groups G1–G6), the morphology of the hBFP-ADSCs was spindle-shaped with an expanded cell cytoplasm, while the hPDLSCs were slim fibroblast-like cells with limited expansion of the cell cytoplasm and prominent cell bodies, and the hDPSCs were spindle-shaped with short and sturdy cell bodies. In the SS (Groups G7–G9), regardless of the cell source, the expansion of the cell cytoplasm was decreased. The morphology of the MSCs became a homogenous population of elongated spindle-shaped cells in the SS. The expanded cells exhibited a homogenous cell morphology of dense, elongated, spindle-shaped cells (Figure 2).

**Figure 3:**
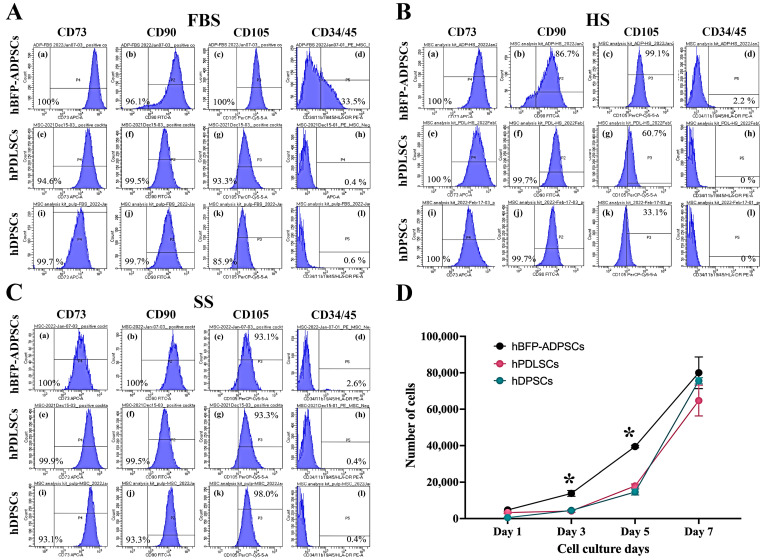
Flow cytometry analysis of mesenchymal stem cell surface antigens and growth of mesenchymal stem cells (MSCs) from the oral cavity, human buccal fat pad-derived adipose stem cells (hBFP-ADSCs), periodontal ligament stem cells (hPDLSCs), and dental pulp stem cells (hDPSCs) in fetal bovine serum (FBS) (**A**), human serum (HS) (**B**), and synthetic serum (SS) (**C**). (**A**–**C**) Flow cytometry of cell surface antigens of mesenchymal stem cells (MSCs), namely, CD73 (**a**,**e**,**i**), CD90 (**b**,**f**,**j**), and CD105 (**c**,**g**,**k**), and the hematopoietic stem cell surface antigen cocktail, namely, CD34, CD11b, CD19, CD45, and HLA-DR (**d**,**h**,**l**), in (**A**) FBS, (**B**) HS, and (**C**) SS; and (**D**) cell proliferation profiles of MSCs in FBS. Symbols: * indicates significantly higher than other groups on days 3 and 5 at *p* < 0.05 (*n* = 3–5, mean ± SD).

Regarding the expression levels of MSC surface antigens, the levels of CD73 and CD90 on all three stem cell sources in FBS, HS, and SS (Groups 1m–9m) were greater than 90% (97.9 ± 3.7%). The expression levels of CD105 tended to vary and be lower than those of other markers, particularly hPDLSCs and hDPSCs in HS (Groups 5m and 6m). The lowest expression level of CD105 at 33.1% was found on hDPSCs in HS (Group 6m). Then, in the SS (Groups 7m–9m), the expression levels of all three markers were greater than 93% (94.8 ± 2.48%) in every group. Notably, the expression levels of the hematopoietic stem cell markers of the hBFP-ADSCs in FBS (Group 1m) were greater than 2% and reached 33.5% (Figure 3A–C). Furthermore, in FBS, the MSCs from different sources (Groups 1m–3m) exhibited different cell proliferation rates; on Days 3 and 5, hBFP-ADSCs (Group 1m) exhibited significantly higher cell proliferation rates than hPDLSCs and hDPSCs (Groups 2m and 3m) (Figure 3D). 

### 3.2. Effects of Stem Cell Sources on Osteogenic Differentiation in Synthetic Serum

**Figure 4:**
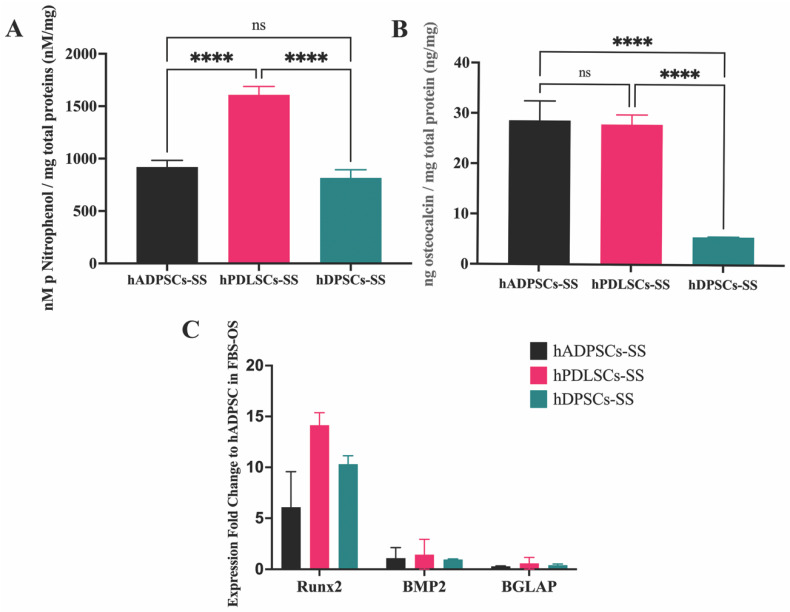
Effects of synthetic serum on the growth and osteogenic differentiation of mesenchymal stem cells from the oral cavity in osteogenic media supplemented with fetal bovine serum (FBS) and synthetic serum (SS). (**A**) Levels of alkaline phosphatase activity (ALP) and (**B**) osteocalcin in culture medium; (**C**) expression fold changes in osteoblast-associated genes, Runx2, bone morphogenetic protein 2 (BMP2), and bone gamma-carboxyglutamate protein (BGLAP) in hBFP-ADSCs in FBS, HS, and SS. Symbols: **** represents significant differences at *p* < 0.001 and ns, >0.05 (*n* = 3–5, mean ± SD).

The synthetic serum promoted osteogenic differentiation of the MSCs from the oral cavity. SS promoted the growth and osteogenic differentiation of MSCs from the oral cavity, hBFP-ADSCs, hPDLSCs, and hDPSCs (Groups 7m–9m), and the levels of osteogenic differentiation of MSCs from different sources in the SS were different. On cell culture plates, the levels of ALP activity in hPDLSCs (Group 8m) were significantly higher than those in hDPSCs (Group 9m) and hBFP-ADSCs (Group 7m) (*p* < 0.01) (Figure 4A), while the levels of osteocalcin in the culture medium of hBFP-ADSCs (Group 7m) and hPDLSCs (Group 8m) were similar (*p* > 0.05) and significantly higher than those in hDPSCs (Group 9m) (*p* < 0.01) (Figure 4B). The promoting effect of SS on an early stage of osteoblastic differentiation of cells from three sources was demonstrated by the upregulation levels of the Runx2 genes of hBFP-ADSCs, hPDLSCs, and hDPSCs in SS (Groups 7m–9m); the expression levels of the Runx2 gene were two-fold greater than the hBFP-ADSCs in the osteogenic medium with FBS (Group 1m). The expression levels of BMP2 and BGLAP in all groups (Groups 7m–9m) exhibited less than a two-fold change (Figure 4C).

### 3.3. Effects of Sera on Growth and Osteogenic Differentiation

**Figure 5:**
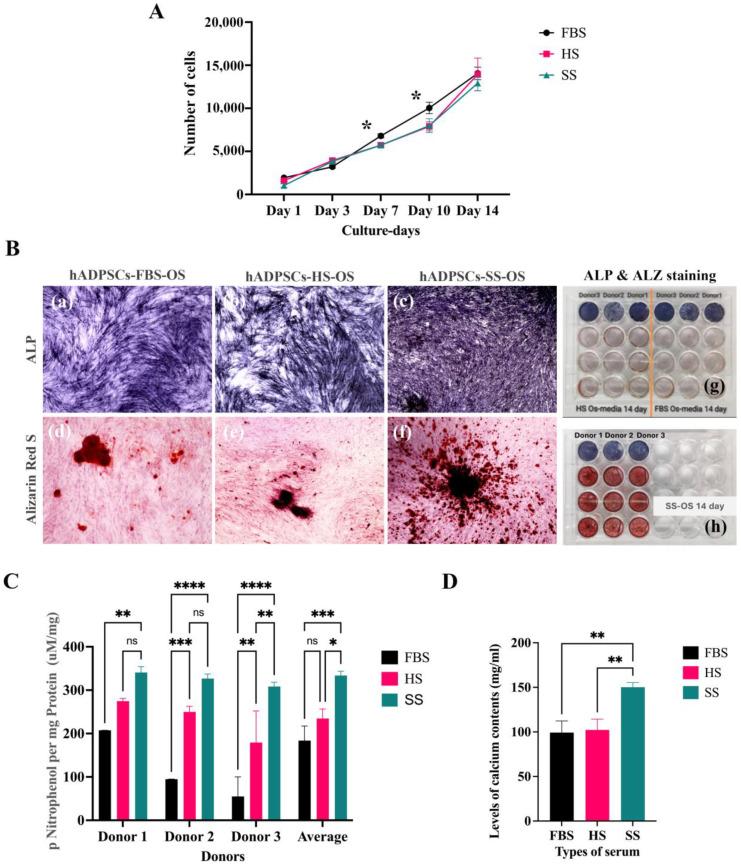
Effects of sera on the growth and osteogenic differentiation of mesenchymal stem cells from the oral cavity represented by human buccal fat pad-derived adipose stem cells (hBFP-ADSCs) in fetal bovine serum (FBS), human serum (HS), and synthetic serum (SS). (**A**) Cell proliferation profiles in growth media; (**B**) alkaline phosphatase (ALP) in blue (**a**–**c**,**g**) and alizarin red (ALZ) staining in red (**d**–**f**,**h**) (magnification ×10); and (**C**) levels of ALP activity and (**D**) calcium content in osteogenic media (OS). Abbreviations: Average, average values from the three donors; Day represents cell culture days; Donors 1–3, donors of the hBFP. Symbols: * indicates significant differences at *p* < 0.05, **, <0.01, ***, <0.001, ****, <0.0001, and ns, >0.05 (*n* = 5, mean ± SD).

The synthetic serum promoted osteogenic differentiation of the hBFP-ADSCs on the cell culture plate but not the encapsulated cells. The effects of serum, FBS, HS, and SS on cell growth and osteogenic differentiation were investigated in hBFP-ADSCs in the osteogenic medium. On the cell culture plate, the levels of cell growth in FBS (Group 1m) on Days 7 and 10 were significantly higher than those in HS (Groups 4m) and SS (Groups 7m) (*p* < 0.05) (Figure 5A). For osteogenic differentiation, the promoting effects of SS on the early and late osteogenic differentiation of hBFP-ADSCs (Group 7m), a representative of MSCs from the oral cavity, were greater and more consistent than those of HS and FBS (Groups 4m and 1m). A higher level of osteogenic differentiation in the SS group (Group 7) than in the HS and FBS groups (Groups 4m and 1m) was demonstrated using intense blue and red staining of ALP and alizarin red staining in the SS group (Group 7m) (Figure 5(Bc,Bf,Bh)). In the FBS and HS groups (Groups 1m and 4m), the levels of ALP activity were not consistent among the three donors, while in the SS group (Group 7m), the levels of each donor were comparable and constantly higher than those in the FBS group (Group 1m) (Figure 5C). The levels of ALP activity and calcium content in the SS group (Group 7m) were significantly higher than those in the FBS and HS groups (Groups 1m and 4m) (Figure 5C,D). 

### 3.4. Effects of the Hydrogel Cell Encapsulation Model on Growth and Osteogenic Differentiation

**Figure 6:**
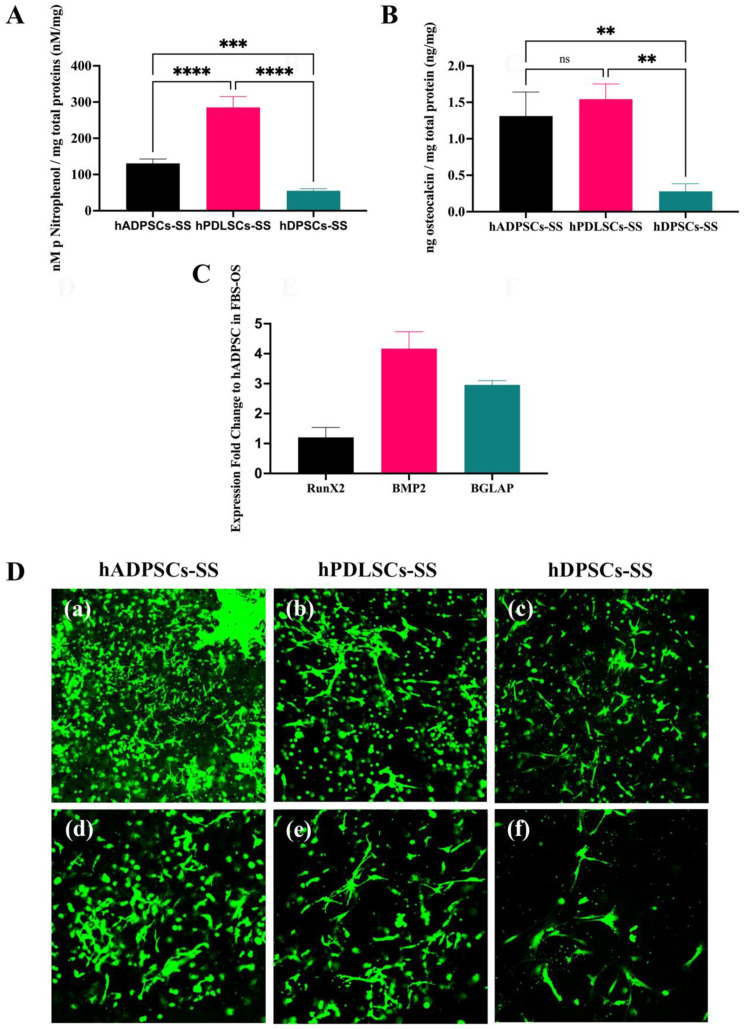
Effects of stem cell sources from the oral cavity on the growth and osteogenic differentiation of encapsulated mesenchymal stem cells in synthetic serum (SS). Human buccal fat pad-derived adipose stem cells (hBFP-ADSCs), periodontal ligament stem cells (hPDLSCs), and dental pulp stem cells (hDPSCs) were encapsulated in the hydrogel and cultured in osteogenic media with the SS. (**A**) Levels of alkaline phosphatase (ALP) activity and (**B**) osteocalcin in the culture medium of the hBFP-ADSCs, hPDLSCs, and hDPSCs; (**C**) expression fold changes in osteoblast-associated genes, Runx2, bone morphogenetic protein 2 (BMP2), and bone gamma-carboxyglutamate protein (BGLAP) of the encapsulated hBFP-ADSCs; and (**D**) confocal laser scanning microscopy of the live/dead cell staining assay of the encapsulated cells in osteogenic medium supplemented with synthetic serum (SS), (**a**,**d**) hBFP-ADSCs, (**b**,**e**), hPDLSCs, and (**c**,**f**) hDPSCs. Magnification ×4 (**D**(**a**–**c**)) and ×10 (**D**(**d**–**f**)). Symbols: ** indicates significant differences at *p* < 0.01, ***, <0.001, ****, <0.0001, and ns, >0.05 (*n* = 3–5, mean ± SD).

**Figure 7:**
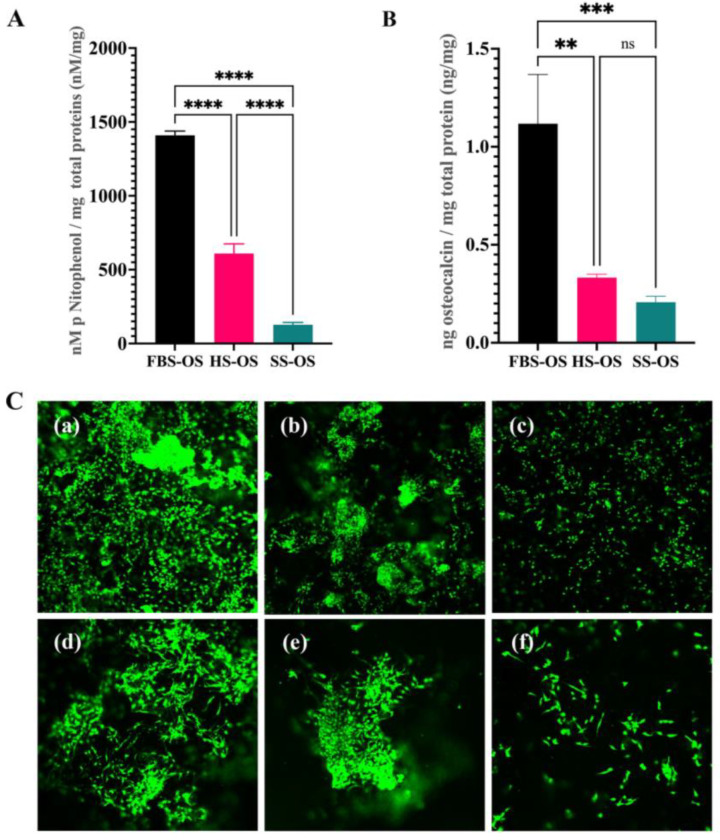
Effects of serum on the growth and osteogenic differentiation of encapsulated human buccal fat pad-derived adipose stem cells (hBFP-ADSCs) in osteogenic media (OS) supplemented with fetal bovine serum (FBS), human serum (HS), and synthetic serum (SS). (**A**) Levels of alkaline phosphatase activity and (**B**) osteocalcin. (**C**) Confocal laser scanning microscopy of the live/dead cell staining assay exhibiting calcein green staining of live cells (**a**,**d**) in FBS, (**b**,**e**) HS, and (**c**,**f**) SS. Magnification 4× (**C**(**a**–**c**)) and 10× (**C**(**d**–**f**)). Symbols: ** indicates significant differences at *p* < 0.01, ***, <0.001, ****, <0.0001, and ns, >0.05 (*n* = 5, mean ± SD).

Regarding the effects of stem cell sources on the encapsulated cells in SS, the osteogenic differentiation potential of the encapsulated cells in SS (Groups 7e–9e) was lower than that of the cells in a monolayer cell culture (Groups 7m–9m) (Figure 5 and Figure 6). Similar to a monolayer cell culture in SS, the encapsulated hPDLSCs (Group 8e) exhibited the highest levels of ALP activity, but in the encapsulated cells, the ALP levels of hBFP-ADSCs (Group 7e) were significantly higher than those of hDPSCs (Groups 9e) (*p* < 0.01) (Figure 6A). Additionally, the levels of osteocalcin in the culture media of the hBFP-ADSCs and hPDLSCs (Groups 7e and 8e) were not significantly different (*p* > 0.05), but were significantly higher than those in the hDPSCs (Group 9e) (*p* < 0.01) (Figure 6B). However, despite the limited levels of osteogenic differentiation of the encapsulated cells in the SS (Groups 7e–9e), the hydrogel encapsulation in SS promoted osteoblastic differentiation into a mature stage. The expression levels of the BMP2 and BGALP genes in the encapsulated hBFP-ADSCs in SS (Group 7e) were upregulated and three- to four-fold greater than those in the hBFP-ADSCs on the cell culture plate in FBS (Group 1m) (Figure 6C). The limited osteogenic differentiation of the encapsulated cells (Groups 7e–9e) was supported by the live/dead staining assay that demonstrated high levels of calcein green staining of viable cells dispersed in the hydrogel, but many cells were round. Few cells could extend their cytoplasm and filopodia to form spindle-shaped cells and intercellular contacts (Figure 6D). In comparison to the cell growth and morphology in FBS (Group 1e) (Figure 7C), the expansion of the cell cytoplasm and intercellular contact of the encapsulated cells in SS were limited (Figure 6D).

Regarding the effects of sera on a cell encapsulation model, the promoting effect of FBS and HS on the growth of the encapsulated cells (Groups 1e, 4e and 7e) was further demonstrated. The SS promoted osteogenic differentiation of the hBFP-ADSCs on the cell culture plate (Group 7m) (Figure 5) but not the encapsulated cells (Group 7e) (Figure 7). The osteogenic differentiation of the encapsulated hBFP-ADSCs in the SS (Group 7e) was significantly lower than that in the FBS (Group 1e) and HS (Group 4e). The levels of ALP activity paralleled the levels of osteocalcin, and the levels in the SS group (Group 7e) were significantly lower than those in the HS (Group 4e) and FBS groups (Group 1e) (*p* < 0.05) (Figure 7A,B). Furthermore, a live/dead cell staining assay exhibited high levels of cell growth and density of viable cells on the hydrogel. Green calcein AM staining showed a high level of cell viability and cell growth in the hydrogel cell encapsulation models, while red staining of nonvital cells was not observed. In FBS and HS (Groups 1e and 4e), the encapsulated cells spread and extended their cytoplasmic process, exhibiting spindle-shaped cells and establishing intercellular contact on the three-dimensional matrix of the hydrogel (Figure 7(Ca,Cb,Cd,Ce)). In contrast, in the SS (Group 7e), the encapsulated cells grew at a low cell density. The spreading of cell cytoplasm on the hydrogel was limited, and the cells were small spindle-shaped cells (Figure 7(Cc,Cf)).

### 3.5. Effects of Sera on the Synthesis of an Extracellular Matrix and Cell Adhesion Molecules of the Encapsulated Cells

**Figure 8:**
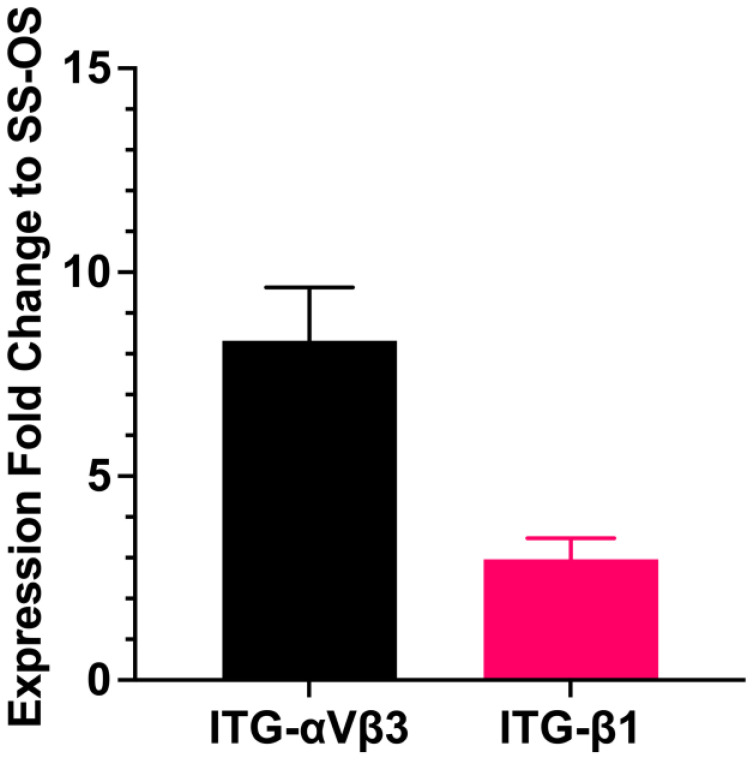
Effects of synthetic serum on the expression of adhesion molecules on hydrogel-encapsulated hBFP-hADCs in synthetic serum (SS-OS) and fetal bovine serum (FBS-OS). Quantitative real-time reverse-transcription polymerase chain reaction (qt-RT-PCR) exhibiting the expression fold change of alpha 5 beta 3 (ITG-αVβ3) and beta 1 (ITG-β1) integrins in FBS-OS relative to those of the SS-OS.

The growth and osteogenic differentiation of the encapsulated cells in SS (Groups 7e–9e) could be affected by the limited cell adhesion of the encapsulated cells in SS. The limitation of cell adhesion was demonstrated by the small and round cells of the encapsulated cells in SS (Figure 6), which was different from the spindle-shaped cells with the extension of cell cytoplasm to establish intercellular contact in FBS and HS (Groups 1e and 4e) (Figure 7). qRT-PCR and immunofluorescence staining demonstrated a decrease in the expression levels of cell adhesion molecules and an ECM protein on the encapsulated cells in the SS-OS group (Groups 7e) compared to the FBS-OS group (Group 1e). The fold changes in the expression of ITG-αVβ3 and ITG-β1 in FBS-OS (Group 1e) were 8.32 ± 1.25 and 2.96 ± 0.46 times higher than those in SS-OS (Group 7e), respectively (Figure 8). The decrease in cell adhesion in SS-OS was further supported through the immunofluorescence staining, showing that the levels of an ECM protein, fibronectin (FN, red), and ITG-αVβ3 and ITG-β1 (green) in SS-OS (Group 7e) were weaker than those in FBS-OS (Group 1e). A summary of the results is shown in Table 2.

## 4. Discussion

The current findings supported the use of hPDLSCs and hBFP-ADSCs as sources of MSCs for cell expansion and transplantation in serum-free conditions to promote bone regeneration. However, based on the availability and accessibility of tissue in all age groups, hBFP-ADSCs exhibited greater applicability than hPDLSCs for clinical applications. Synthetic serum promoted the expression of MSC surface antigens and the growth and osteogenic differentiation of MSCs, and decreased variations between donors. A three-dimensional structure of the thermosensitive nHA–CaCO_3_–chitosan/collagen hydrogel promoted the osteogenic differentiation of MSCs in FBS and SS. The findings highlighted that low levels of adhesion molecules in the SS were attributed to poor cell adhesion, growth, and osteogenic differentiation of the hydrogel-encapsulated cells in the SS cell culture.

As MSC biological behaviors can vary with the different synthetic serum formulas [16,20], the results from the current study were reported based on the use of the selected serum-free synthetic culture medium (StemPro™ MSC SFM, Thermo Fischer, USA) in the SS group. The SS was used to determine the optimal stem cell sources from the oral cavity for serum-free cell expansion and encapsulation for bone regeneration. All MSCs from the three sources in the SS cell culture exhibited comparative properties similar to the cells in FBS [29], as the osteogenic differentiation potential of hADSCs and hPDLSCs was comparable and higher than that of hDPSCs, even though they exhibited homogenous elongated spindle-shaped cells in the SS. The differences between stem cell sources were further underlined by a greater proliferation rate of the hBFP-ADSCs than the hPDLSCs and hDPSCs. It could be hypothesized that the different cell morphologies [53] and unique local microenvironments of stem cell sources, such as vascularization, cytokines, and host cells, could contribute to the different stem cell behaviors [41,54,55]. Additionally, hBFP-ADSCs were isolated from the hBFP, which was obtained during the routine surgical removal of the upper third molars that caused minimal discomfort to the donors. On the other hand, the dental pulp and periodontal ligament were harvested from the extracted teeth. Therefore, the availability of tissues is another factor contributing to the MSC of choice for transplantation.

Regarding the effects of SS on MSC characteristics and the biological behaviors of MSCs from three sources in the oral cavity, the findings agreed with previous reports that MSCs are spindle-shaped cells with variations in cell size and shape [43,56,57]. The homogenous elongated spindle-shaped cell morphology of MSCs in the SS could create a uniform cell attachment and focal contact signal transduction that standardized osteogenic differentiation potential among donors [53,58]. Moreover, the SS promoted the expression of the characteristic surface antigens of MSCs, namely, CD73, CD90, and CD105, to levels higher than 90% of the total cell population. Low expression levels of CD105 on hDPSCs in FBS and HS could indicate a deficient chondrogenic differentiation potential of MSCs [59,60]. Additionally, the high level of hematopoietic stem cell surface antigens found in the hBFP-ADSCs in FBS could be because the hBFP-ADSCs were derived from vascular stromal cells [61]. Thus, transplanted hADSCs would be able to enhance new vascularization and bone regeneration at transplantation sites [62,63]. Variations in the levels of CD105 and hematopoietic stem cell markers, and the cell growth of MSCs in FBS suggest a heterogeneous cell population and different stemness of MSCs from different sources [28,64]. Together with the homogenous cell morphology and high levels of MSC surface antigens in the SS, it could be hypothesized that the standardization and promoting effects of the SS would improve the consistency and predictability of treatment outcomes of stem cell transplant and facilitate allogenic stem cell transplantation. 

It was clearly shown that the osteogenic differentiation potential of MSCs was influenced by the types of sera [14,16,17,20] and sources of stem cells [64,65]. Osteoblastic differentiation in the early and late stages was represented by the expression levels of Runx2 genes and ALP activity, the expression levels of BMP2 and BGLAP genes, and the levels of in vitro mineralization and osteocalcin, respectively [51,52]. As it was found that in a monolayer cell culture, the levels of the early and late osteoblastic differentiation markers in the SS were higher than those in FBS, the findings agreed with previous studies that the SS promoted osteogenic differentiation of the MSCs into a mature stage and functional osteoblasts [18,19,20]. Based on the levels of osteocalcin and in vitro mineralization in the SS, the osteogenic differentiation potential of the hADSCs and hPDLSCs was comparable and higher than that of the hDPSCs. Considering the availability of adipose tissue and cells, hBFP-ADSCs were selected for further investigations of the effects of serum and cell culture models. The advantages of hBFP as a stem cell source for promoting bone regeneration are supported by previous studies. hBFP transplantation promotes the amount and rate of bone regeneration [27,32,66]. The BFP that is transplanted with an inorganic bovine bone mineral [10,11], autogenous ramus cortical plates [10], and an amniotic membrane could successfully reconstruct atrophic and pathological maxillary-mandibular ridges and defects, respectively [27]. Based on the availability and accessibility of the tissue throughout life and in all age groups via minimally invasive surgical procedures [11,30,66], hBFP-ADSCs are a viable source of hMSCs for regeneration.

Regarding the hydrogel cell encapsulation model, the high level of cell viability and osteogenic differentiation potential of the encapsulated MSCs support previous studies that the hydrogel was noncytotoxic and a three-dimensional structure and osteoconductive matrix of the in-house thermosensitive nHA-CaCO_3_-chitosan/collagen hydrogel could promote cell growth and osteogenic differentiation of the encapsulated cells [35,37,38]. Despite a compromised condition in the SS, the hydrogel could still promote osteoblastic differentiation of the MSCs into a mature stage, as indicated by upregulation of the BMP2 and BGLAP genes of the encapsulated cells that were not found in the monolayer cell culture in the SS [51,52]. Additionally, the findings highlighted the supporting microenvironment of the thermosensitive nHA–CaCO_3_–chitosan/collagen hydrogel for cell growth and osteogenic differentiation of the MSCs [30,51] and suggested that the thermosensitive nHA–CaCO_3_–chitosan/collagen hydrogel cell encapsulation would support stem cell transplantation into the defect sites [5,67,68]. Furthermore, the thermosensitive property and injectability of the hydrogel [37] would enable a delivery of stem cells into difficult-to-reach areas and irregular-shaped skeletal defects through a minimal surgical exposure [67,69] and three-dimensional printed scaffold fabrications for a customized alveolar bone augmentation [5]. Therefore, it would increase efficiency, safety, and patient comfort in alveolar bone regeneration. Additionally, an ability of the hydrogel to deliver bioactive molecules [37,38] would further enhance the ability of the hydrogel to regulate stem cell fates and promote the regenerative capacity of the encapsulated cells in the defect sites [12,34]. 

A small, contracted, and spherical cell shape of the encapsulated cells In the SS contrasted with the morphology of the encapsulated cells in the FBS and HS, wherein they spread out and established an intercellular network. The findings indicated poor cell adhesion that corresponded with a lack of the promoting effects of the hydrogel in the SS and a lack of ECM and adhesion molecules of the encapsulated cells in the SS [70,71]. This could be because ECM and adhesion molecules, such as fibronectin, ITG-αVβ3, and ITGβ1, are important molecules regulating the stemness, adhesion, and osteogenic differentiation of MSCs on the three-dimensional structure of contact-dependent MSCs [60,71,72]. As it has been reported that surface coating and cell adhesion are important regulating factors for successful cell culture in serum-free cell culture [70,73,74], the limited cell spreading and low levels of adhesion molecules of the encapsulated cells in SS possibly contributed to the low osteogenic differentiation potential of the encapsulated cells in the SS. The findings agreed with previous reports that FBS is an essential source of adhesion molecules regulating stem cell behaviors [75] and highlight the important roles of cell adhesion molecules, such as ITG-αVβ3 and ITG-β1, as key factors regulating the growth and differentiation of hydrogel-encapsulated MSCs. Although it could be expected that the amount of cell adhesion molecules could be increased when the hydrogel contacts human blood in the defect sites [68], further study should improve the expression of adhesion molecules and ECM on the hydrogel matrix to enhance the efficiency of stem cell encapsulation and delivery in serum-free conditions.

## 5. Conclusions

The findings of this study demonstrated that a proposed nanohydroxyapatite–calcium carbonate microcapsule–chitosan/collagen hydrogel was applicable for stem cell encapsulation and delivery in serum-free conditions, and human buccal fat pad-derived adipose stem cells were the optimum stem cell source for stem cell encapsulation and transplantation. Additionally, synthetic serum standardized the quality of mesenchymal stem cells (MSCs) and promoted the osteogenic differentiation potential of MSCs from the oral cavity, where hPDLSCs and hADSCs were superior to hDPSCs. The low levels of integrin beta 1 (ITG-β1), and integrin alpha 5 beta 3 (ITG-α5β3) adversely affected the growth and osteogenic differentiation of the hydrogel-encapsulated cells. Further study should be performed to improve the cell adhesion of the encapsulated cells in serum-free conditions and investigate the bone regeneration of the encapsulated hBFP-ADSCs in skeletal defects in an animal model.

## Figures and Tables

**Table 1:** Study groups.

	Sets I/III	hBFP-ADSCs		hPDLSCs		hDPSCs	
Set II		Mono	Encap	Mono	Encap	Mono	Encap
FBS		Group 1m	Group 1e	Group 2m	-	Group 3m	-
HS		Group 4m	Group 4e	Group 5m	-	Group 6m	-
SS		Group 7m	Group 7e	Group 8m	G8e	Group 9m	Group 9e

Abbreviations: Set I, stem cells from different cell sources; Set II, type of sera; Set III, cell culture models; hBFP-ADSCs represent buccal fat pad derived adipose stem cells; hPDLSCs, human periodontal ligament stem cells; hDPSCs, human dental pulp stem cells; FBS, fetal bovine serum; HS, human serum; SS, synthetic serum; Mono and m, mono layer cell culture; Encap and e, hydrogel cell encapsulation.

**Table 2:** Summary of cell culture conditions, investigations, and findings.

Cell Culture Conditions (Study Groups)	Investigated Parameters	Findings
SC sources - hADSCs, hPDLSCs and hDPSCs Types of sera - FBS, HS and SS	MSC characteristics - Morphology - MSC surface antigens (CD73, CD90 and CD105)	- SS decreasing differences MSCs from different sources - Homogenous spindle-shaped cells and increased expression levels of surface antigens
Effects of SC sources in the SS	OS differentiation potential - Mono layer	- hPDLSC-hADSCs >>> hDPSCs
	- Hydrogel encapsulation	- hPDLSC-hADSCs >>> hDPSCs
Effects of Types of Sera (FBS and SS) on hADSCs	OS differentiation potential - Mono layer cell culture	- Decreasing effects of donor variations ○ SS >>> FBS
	- Hydrogel encapsulation	- 3D >>> 2D - FBS >>> MSC
	Levels of adhesion molecules - FN, ITGs	- Decreasing in SS - FBS >>> SS

Abbreviations: SC, stem cells; hADSCs, human buccal fat pad (hBFP)-derived adipose stem cells; hPDLSCs, periodontal ligament stem cells; hDPSCs, dental pulp stem cells; FBS, fetal bovine serum; SS, synthetic serum; FN, fibronectin; and ITGs, integrins. Symbols: -, equal or similar levels to; and >>>, significantly higher than.

## Data Availability

The data of this study are available from the corresponding author upon a reasonable request.
